# Pleiotropic effects of Apolipoprotein (ApoE) 4 on executive function in mice and humans

**DOI:** 10.1002/alz70855_099431

**Published:** 2025-12-23

**Authors:** Madison R Longmuir

**Affiliations:** ^1^ Schulich School of Medicine & Dentistry, London, ON, Canada; Robarts Research Institute, London, ON, Canada; University of Western Ontario, London, ON, Canada

## Abstract

**Background:**

ApoE4 is the strongest genetic risk factor for late‐onset Alzheimer's disease (AD). Compared to ApoE4 noncarriers (ApoE4‐), ApoE4 carriers (ApoE4+) exhibit earlier onset and more rapid progression of memory impairment. However, separate lines of work have suggested the opposite pattern in other cognitive domains: ApoE4+ individuals exhibit superior executive function compared to ApoE4‐, but this advantage decreases with age. The antagonistic pleiotropy hypothesis proposes that ApoE4 may confer advantages to brain function at earlier ages, but these advantages become detrimental later in life. To investigate this hypothesis, we took a cross‐species translational approach focusing on cognitively unimpaired human adult ApoE4+ and ApoE4‐, ages 60‐80 years, in combination with knock‐in mouse models that express humanized wild‐type App, MAPT, and ApoE3/4 genes.

**Method:**

To quantify executive function, we utilized the cross‐species touchscreen‐based Continuous Performance Task (CPT) to evaluate selective attention. Human participants were drawn from the PREVENT‐AD program at the Douglas Research Centre, with testing conducted on MS SurfacePros. Mice performed the same task using an operant chamber platform.

**Result:**

Behavioural results suggest that ApoE4 facilitates attentional processes in both older adult and mouse ApoE4 carriers, as indexed by discrimination performance on the CPT. Further strengthening this translational pattern, we found that the facilitative effect of ApoE4 on CPT was more pronounced in female ApoE4+ of both species. In mice, we examined the ApoE‐dependent effects on attention longitudinally (spanning 12 months), finding that the positive effect of ApoE4 was sustained across this interval in females, though it became less robust with age. In contrast, ApoE4 was beneficial in males only during early life. Notably, these effects were observed in the absence of detectable brain pathology

**Conclusion:**

These results support the antagonistic pleiotropy hypothesis, whereby ApoE4 carriership confers greater attentional performance early in life and in the absence of classical AD pathology. This effect is detectable in both sexes but is stronger and more sustained across age in females. All these patterns are conserved across humans and mice, suggesting that this cross‐species translational platform is well suited to explore the mechanistic basis of ApoE4 antagonistic pleiotropy as it relates to domain‐specific cognitive function.

